# Can humanoid robots be used as a cognitive offloading tool?

**DOI:** 10.1186/s41235-025-00616-7

**Published:** 2025-04-17

**Authors:** Shari Cavicchi, Abdulaziz Abubshait, Giulia Siri, Magda Mustile, Francesca Ciardo

**Affiliations:** 1 Social Cognition in Human-Robot Interaction, Italian Institute of Technology, Genoa, Italy; 2 Department of Neuroscience and Rehabilitation, University of Ferrara, Ferrara, Italy; 3 The Psychological Sciences Research Institute, University of Louvain, Louvain-La-Neuve, Belgium; 4 Department of Psychology, University of Milano-Bicocca, Milan, Italy

**Keywords:** Cognitive load, Human–robot interaction, Cognitive offloading

## Abstract

Cognitive load occurs when the demands of a task surpass the available processing capacity, straining mental resources and potentially impairing performance efficiency, such as increasing the number of errors in a task. Owing to its ubiquity in real-world scenarios, the existence of offloading strategies to reduce cognitive load is not new to experts and nonexperts, and many of these strategies involve technology (e.g., using Calendar Apps to remember scheduled events). Surprisingly, little is known about the potential use of humanoid robots for cognitive offloading. We will examine studies assessing the influence of humanoid robots on cognitive tasks requiring the resolution of cognitive conflict to determine whether their presence facilitates or hinders cognitive performance. Our analysis focuses on standardized cognitive conflict paradigms, as these effectively simulate real-life conflict scenarios (i.e., everyday challenges in focusing on the task and ignoring distractions). In these studies, robots were involved by either participating in the tasks, providing social cues, or observing human performance. By identifying contexts where humanoid robots support cognitive offloading and where they may undermine it, this work contributes to a deeper understanding of cognitive processes in human–robot interaction (HRI) and informs the design of interventions aimed at improving task performance and well-being in professional HRI settings.

## Significance

Because of its detrimental effects, cognitive load is a key concept across many research fields, together with the search for strategies to reduce it (i.e., cognitive offloading). We know that technology can be used to reduce load (e.g., we normally use Calendar Apps to remember scheduled events), but we're still figuring out how to maximize the role of humanoid robots in reducing cognitive load for human operators. This paper aims to delve into this knowledge gap, exploring how cognitive processes work during human-robot interaction (HRI), specifically during tasks in which cognitive load arises from cognitive conflict. Addressing how humanoid robots can impact cognitive load during HRI, opens the door to a future where these robots become helpful partners in making work easier. This doesn't just mean improving performance, but it also brings us closer to creating workplaces where people feel less stressed, making it a big leap toward humans and robots working efficiently side by side.

## Introduction

Cognitive load (or workload; Longo et al., 2022) refers to a state of exhaustion of the cognitive resources available at a given moment (e.g., Kahneman, 1973; Lavie, 2010; Musslick & Cohen, 2021). It encompasses the demands placed on an individual's cognitive system by the tasks they are performing (Colombi et al., 2012; Palinko et al., 2010), including the complexity of the information (Stock et al., 2016) and the mental resources required to manage and process this information effectively (Leung et al., 2010; Liang et al., 2014). When individuals are cognitively overloaded, they tend to make more errors (e.g., Thomas et al., 2017), experience increased stress (e.g., Masi et al., 2023), and find it more challenging to learn new skills (e.g., Kirschner, 2002).

Because of its adverse effects, finding solutions to mitigate cognitive load, for example, via cognitive offloading strategies (Risko & Gilbert, 2016), has been crucial to human interests and needs (e.g., Lodge et al., 2023; Nückles et al., 2020). The concept of utilizing offloading strategies to alleviate cognitive load is well established among experts (Risko & Gilbert, 2016, for a review) and nonexperts. For example, we are all familiar with moving our fingers to help with mental arithmetic or noting down a password that we need to remember in a notebook. Thanks to technological innovations, we are also becoming more familiar with the use of new tools for cognitive offloading, such as adding reminders on calendar apps to help prospective memory for all the scheduled appointments (McDonald et al., 2011). However, what is less clear is the role played by social artificial agents, such as humanoid robots, on the cognitive load experience in a given task, as the potential use of humanoid robots for cognitive offloading is yet to be understood. Like any technological tool, they are developed and designed to help humans, but their social nature may have adverse effects on cognitive load in human users they interact with.

This paper aims to delve into this knowledge gap, describing the current evidence and elucidating when humanoid robots can optimize performance during cognitive tasks or be detrimental to it. We believe that, with the right setting, humans can benefit from cooperative and competitive scenarios shared with robots. To do so, the current review is organized as follows: we first review evidence from human–human interactions by focusing on cognitive load experienced in well-established cognitive conflict tasks, as these effectively simulate real-life conflict scenarios like staying focused on the task while ignoring distractions. Second, we focus on studies that explored the role of humanoid robots acting as teammates, delivering social cues, or just observing the human user performing a cognitive task. We intentionally focused on studies that investigated the influence of human–robot interaction (HRI) on standardized cognitive conflict paradigms, such as the Flanker task (Eriksen & Eriksen, 1974), the Stroop task (Macleod, 1991; Stroop, 1935), the Simon task (Simon & Rudell, 1967), the stop-signal task (Logan et al., 1984), or the go/no-go task (Donders, 1868; 1969), see Table 1 for a description of such paradigms. Although non-standard paradigms might offer valuable insights, cognitive conflict paradigms provide a clearer lens through which to examine the cognitive processes underlying specific activities, facilitating a broader generalization of the findings. Finally, we will highlight what precautions should be considered when designing both the robots and the shared environments (e.g., protocols aimed at integrating robots into daily routines), and we introduce general recommendations that could help researchers advance the theoretical and applied knowledge in HRI, by supporting the reliability and replicability of results.

**Table 1:** Short description of the main tasks used to study cognitive conflict in cognitive sciences

Task	Description
Eriksen Flanker task (Eriksen & Eriksen, 1974)	The observers must attend to a location where they know a target is going to appear. The target is taken from a set of two possible items, each one requiring a specific response. The participant is instructed to recognize and indicate the target’s identity. Critically, the target is flanked by other items, that could be either mapped to the same response associated with the target (congruent trials), with the opposite response (incongruent trials), or with no response (neutral trials). What is usually observed, is that RTs are slower in the presence of letters that are mapped to an incongruent response rather than the congruent one (congruency effect). The conflict here is caused by the interference of the non-target’s identity in the selection of an appropriate response because they activate incorrect responses if attended to (e.g., Lleras et al., 2013)
Stroop task (Macleod, 1991; Stroop, 1935)	The participants have to name the color in which a word is displayed. Different colors are typically associated with different responses. The meaning of the written word could either match the color in which the word is written (congruent trials; e.g., the word “green” colored in green) or another color (incongruent trials; e.g., the word “yellow” displayed in green). The typical finding is that observers’ performance is impaired when the color and the written word are not matching, and thus mapped with conflicting responses, compared to trials in which the color and the word are matching
Simon task (Simon & Rudell, 1967)	The Simon task is used to investigate the spatial compatibility effect for a task-irrelevant stimulus location. Participants are required to respond to a nonspatial feature (e.g., color or shape) of a stimulus presented to the left or right of fixation with a right or left key press, ignoring its location. Performance is faster and more accurate when stimulus and response locations spatially correspond compared to when they do not correspond (e.g., the Simon effect), hence indicating that stimulus location affects performance even if task-irrelevant. It is widely assumed that the Simon effect is due to a conflict, emerging at the stage of response selection (e.g., Rubichi et al., 2000) between two alternative response codes, one generated based on task instructions and the other automatically activated through preexisting associations linking a stimulus to its spatially corresponding response
Stop-signal task (Logan, Cowan, & Davis, 1984)	Participants have to perform a two-choice reaction time task, prompted by a “go” signal. In a minority of trials, a stop-signal stimulus (e.g., a sound) is presented at a varying delay after the “go” signal, to inform the participants they have to withdraw the response they are preparing (stop trials). The delay between the go and the stop signal (stop-signal delay; SSD) can be a priori decided or adjusted online based on the performance of the participant. Typically, decreasing the delay makes the stop trial easier and increases the probability of success, while increasing the delay does the opposite. The stop-signal task allows us to estimate the time needed to withhold an action that is already initiated (i.e., stop-signal reaction time; SSRT)
Go/no-go task (Donders, 1868; 1969)	In a go/no-go task, participants are required to respond to specific stimuli (go trials) and withhold a response to others (no-go trials). The primary measure is the rate of commission errors (CE; i.e., the proportion of responses that were not successfully withheld). A high proportion of CE indicates poor response inhibition. Two other variables are typically derived: correct detection reaction times (CRT) and omission errors (OE; i.e., failures to respond to a go stimulus), which are thought to reflect lapses of attention or vigilance

## Humans, cognitive load, and social interactions

In psychology literature, the term “cognitive load” has been attributed to task demands (e.g., Colombi et al., 2012; Palinko et al., 2010), self-experienced effort to fulfill goals (Baldauf et al., 2009), the amount of memory resources used to perform a task (Stack et al., 2016), an individual’s processing capacity in use at a given moment (Young et al., 2008), the attentional resources needed to make decisions (Young & Stanton, 2001; Young et al., 2015), or a combination of mental effort, information processing and emotional response to a task (e.g., Miller, 2001; Pierce, 2009; Sheridan & Simpson, 1979; see Longo et al., 2022, for a review). We will not list the definitions and models that have been used in the literature to describe cognitive load since it has already been done in a recent comprehensive review (Longo et al., 2022), but we specify that throughout this manuscript we refer to cognitive load as *“the relative demand imposed by a particular task, in terms of mental resources required”* (American Psychological Association, n.d.). Thus, for the current work, we refer to the effort required to complete a task, encompassing the effort needed in both single- and multi-tasking scenarios.

Several studies showed that individual performance in simple tasks, such as in cognitive conflict paradigms listed in Table 1, is affected by the presence of other humans. For instance, evidence showed that the mere presence of others could result in faster and more accurate performance (Aiello & Douthitt, 2001), an effect known as "social facilitation" that could be due to an increase in arousal elicited by the presence of another human being (Guerin, 1999). Similar results have also been reported by studies investigating perception–action interference. In a series of experiments, Brass and colleagues (2000) tested the effect of observing another human performing the same task on finger movement execution. Results showed that action execution was more accurate and faster when observing someone else performing the same action, compared to when observing an opposite action (Brass et al., 2000, 2001; Kilner et al., 2003). Whan and colleagues (2017) investigated the effect of task sharing on cognitive load in a visual search task. Pairs of participants were asked to track target objects among moving distractors while their eye movements were recorded. The task was performed jointly, alone, or in pseudo-pairs (two participants performing the task independently). Performance was better in the joint condition compared to the individual and the pseudo-pair ones. Critically, eye movements analysis revealed that participants used a division of labor strategy (i.e., each participant monitoring targets on only one side of the monitor; see also “Guidelines for the use of humanoid robots for cognitive offloading”, guideline 4).

Notably, task sharing with others is not always beneficial in cognitive conflict tasks. Cardellicchio and colleagues (2021) showed how the presence of a human partner in a stop-signal task (Logan et al., 1984; see Table 1) can negatively influence response inhibition. The aim of the study was to investigate the role of response inhibition in a joint action (JA) context. To this purpose, they instructed participants to perform a go task, where participants were asked to open a bottle held by a clamp (control/solo condition) or held by a human partner (JA condition), and a stop task, where they were asked to suppress that action when a stop sound was delivered. The authors found that in the JA condition, action suppression slowed down, likely indicating the need for representing both agents’ actions, requiring the simultaneous preparation of multiple motor plans and eventually increasing the cost of stopping compared to when acting in solo (Cavallo et al., 2014). Even though participants can functionally integrate information about a human partner’s behavior and adaptively adjust their own responses based on that, this does not lead to any performance facilitation (see also Sebanz et al., 2003; Ferraro et al., 2011 for similar results on a joint Simon task). In sum, evidence addressing the role of task sharing among humans as an offloading strategy to reduce cognitive conflict has yielded mixed results, with some studies highlighting how social interaction can have detrimental effects on performance in cognitive tasks. An intriguing open question refers to whether or not interacting with social artificial agents (e.g., Hertz & Wiese, 2019), such as humanoid robots, similarly affects cognitive load.

## Technology, humanoid robots, and cognitive offloading

In developing society 5.0 (Roblek et al., 2020; a futuristic society that integrates technology and AI), external aids, such as apps for smartphones (Grinschgl et al., 2023), artificial intelligence (AI) algorithms (Ritz et al., 2024), or robotic or computer agents (Hertz & Wiese, 2019), can be used to alleviate cognitive load, thereby facilitating the completion of otherwise challenging tasks. The delegation of either tasks or information storage in external resources is referred to as cognitive offloading (Risko & Gilbert, 2016), which is crucial for freeing up cognitive resources, enabling more effective information processing, and eventually enhancing productivity (e.g., Clark, 2011; Kirsh, 2010; O’Hara & Payne, 1998).

Throughout human evolution, the creation and use of tools have been pivotal in supporting humans in their daily routines. An example is represented by robotic agents. Many studies have investigated the efficacy of HRI, not only in terms of reduced physical effort (e.g., posture or movements; Shugaba et al., 2022) but also in terms of benefits for cognition (e.g., Hertz & Wiese, 2019). For instance, depending on the level of automation of a robotic system, some decisional tasks can be completely allocated to the robotic agent, leading to the perception of fewer responsibilities for the operator, as robots might offload them from duties and work, eventually reducing cognitive load (for a review see, Baltrusch et al., 2022). While the potential of technology as an offloading strategy has been extensively studied (see Risko & Gilbert, 2016, for a review), little is still known about humanoid robots as cognitive offloading tools in situations of cognitive conflict. This is surprising because humans and robots are already working shoulder to shoulder in cooperation in many environments, ranging from health care (Bainbridge, 2008; Kiesler, 2008; Kose, 2015; Wang 2019), education (e.g., Belpaeme et al., 2018), and industry (Liu et al., 2022; Wang et al., 2020; Zhang et al., 2020) to delicate work routines such as surgery (e.g., Shugaba et al., 2022) or rescue operations (Spenko et al., 2018). Understanding the potential of humanoid robots as collaborators and teammates would open promising avenues for cognitive load mitigation and for the optimization of many workplaces.

Humanoid robots are engineered to resemble and move like humans (e.g., they have bodies, and their heads are equipped with "face-like" features, such as movable mouths and eyes; Dautenhahn, 2007; Fong et al., 2003; Marchesi et al., 2024). The presence of human-like features and behaviors in automated agents has been suggested to make humans more prone to consider robots as social entities, improving the acceptance of robots as team partners (Wiese et al., 2017). Moreover, evidence in human–robot interaction has observed the beneficial effects of including robots in teams (e.g., Chen & Barnes, 2014; Walliser et al., 2019). Such benefits range from increased empathy toward the robot (de Jong et al., 2021), reduced stress caused by failures or fear of failing in a task (Strohkorb Sebo et al., 2018), to improved team performance (Chen & Barnes, 2014; Walliser et al., 2019), especially when robots can engage in social dynamics and adapt their behavior to a social environment, i.e., when we refer to them as social agents (robots) instead of simple automated machines (Duffy, 2003). Social robots not only can team up with humans during physical and cognitive tasks (Ciardo & Wykowska, 2022; Wiese et al., 2017; Wykowska, 2021), but also can use social signals, like gaze or eye contact, to express intentions or feelings (e.g., Perez-Osorio et al., 2021; Abubshait et al., 2021c). In the next sections, we will discuss some studies showing the benefits of having humanoid robots included in joint scenarios, and how this affects cognitive load in cognitive conflict tasks. Nonetheless, we will also discuss instances in which interacting with robots can be disadvantageous for humans. We believe that researchers should consider the pros and cons of applying humanoid robots in their work as this type of research sheds light on which robot design features can be helpful in real-life HRI scenarios.

## Humanoid robots in cognitive conflict tasks

### Task sharing with robots

The more intuitive way in which humans can use robots to manage cognitive load is by transferring a task to them or by sharing joint activities (e.g., Abubshait et al., 2021c; Abubshait et al., 2024; Perez-Osorio et al., 2021). Task sharing is the most frequent and spontaneous way humans manage cognitive load when in the presence of another agent, even if it is a robot. Prior research suggested that collaborating with humanoid robots might impact an individual’s behavior similarly to what happens during human–human interactions. Bunlon and colleagues (2018) used a modified version of the Simon task (Simon & Rudell, 1967; see Table 1). The standard Simon task was adapted to a joint virtual scenario (joint Simon task; Sebanz et al., 2003), in which the task was performed together with a virtual human or robotic hand. Participants sat in a room alone, next to an empty chair, and placed one of their hands on a mouse key. A rectangle was displayed on a screen and contained three circles: one at the center of the rectangle, one to the left, and one to the right. Below the rectangle, two hands placed on a mouse appeared. One represented the participant’s own hand, and the other one could be either a human or robotic hand, depending on the condition. Participants were instructed to press the key when one of the circles turned in a target color (i.e., blue or orange), and to withhold any action when a circle turned in the opposite color (that was assigned to the companion). The authors observed a Simon effect (SE, indexing cognitive conflict) when participants completed the task with virtual humans and virtual robots, with no difference in the magnitude of the effect between the two conditions, likely indicating that the SE arises when individuals interact with other agents, regardless of their nature (human or artificial). These findings were replicated in other studies investigating the SE and task sharing with robots across different cultural contexts (Ciardo et al., 2022; Sobel & Sims, 2022; Strait et al., 2020) and provide important insight into how humans can represent a task as shared when interacting with robotic partners. Similarly to what happens during human–human collaboration, a shared representation of the task is created when acting with robots. However, as mentioned above, task demands are not divided but shared, since participants represent the same amount of information they would represent when performing the task alone, and thus performance is not improved by the presence of a robotic partner. The implications of the results observed by Bunlon and colleagues (2018) will be described more extensively in “Guidelines for the use of humanoid robots for cognitive offloading”.

### Robots delivering social signals

Importantly, engaging robots in joint tasks is not the only way robots can affect cognitive conflict. Humanoid robots, as previously said, can use social signals, like gaze or mutual gaze, to gaze at important elements in the field or express intentions to their human companions (Ciardo & Wykowska, 2022; Wiese et al., 2017; Wykowska, 2021). Social signals are important because they provide external cues that can help individuals while performing cognitive tasks, potentially mitigating cognitive load (Hsiao et al., 2012, 2016). For instance, not only do social signals represent additional information sources that can guide an individual’s actions, but they can also be used to convey feelings of social support and encouragement, that can alleviate stress and anxiety (Handley et al., 2012; Leavy, 1983). By interpreting and responding to social signals, individuals can navigate complex social interactions more smoothly, conserving mental resources for other tasks (e.g., Frith & Frith, 2007). The fact that a robot’s social signals can guide humans’ actions, is a double-edged sword. While designing collaborative scenarios, one should keep in mind that social signals could involuntarily divert attention toward irrelevant locations or items. Consistently, Perez-Osorio and colleagues (2021) asked participants to perform a categorization task on some colored objects. These objects were presented on a screen held by a humanoid robot and could be colored either in blue or in yellow, with different percentages of the two colors (e.g., a 100% blue object, a 75% blue and 25% yellow object, an object half yellow and half blue, and the complementary combinations). On each trial, participants categorized the objects as blue or yellow, by selecting one of two bins appearing on the two top corners of the screen. The robot moved its arm as if it was handing the object over to the participant, and then moved its head to cue the correct or incorrect bin with equal probability. Seeing the robot cueing the wrong bin was proposed to induce cognitive conflict, interfering with task performance. In particular, the cognitive conflict would be caused by the mismatch between the robot's social signal and the bin chosen in incongruent trials. In this condition, similarly to classic cognitive conflict tasks (like the Stroop task, Stroop, 1935; or the Flanker task, Eriksen and Eriksen, 1974), interfering information (i.e., the robot's head cue) needed to be ignored. In line with prior literature (e.g., Friesen & Kingstone, 1998), longer reaction times for incongruent head movements of the robot relative to congruent head movements were observed, and more errors were committed when the task was more difficult (i.e., when the percentages of blue and yellow in the colored object were closer to 50%). Critically, these results showed that incongruent social signals from an artificial agent can impair performance in cognitive tasks, even if the social signal is irrelevant to the task. This is important because it shows that social artificial agents' behavior is not always helpful, but could be, instead, distracting. In a similar experiment that used a humanoid robot, Abubshait and colleagues (2024) suggested that the processing of social stimuli might prevail on task-relevant information processing, making the presence of a robot detrimental to task performance.

Eye contact (or mutual gaze) with a robot is another factor that should be considered while designing collaborative HRI scenarios (Kompatsiari et al., 2022). Spatola and Huguet (2021) used a social Eriksen Flanker task (Eriksen & Eriksen, 1974) to examine whether robots’ gaze was interpreted as a social signal during a cognitive conflict task. They asked participants to watch a video of a robot interacting with a human. Afterward, participants completed a Flanker task where they saw a line of arrows (i.e., flankers) pointing to the left. They were instructed to identify whether the middle arrow pointed to the left or to the right. On congruent trials, the flanks pointed in the same direction as the middle arrow, while on incongruent trials, the target arrow pointed in the opposite direction of the flankers. Throughout the Flanker task, a picture of the robot was superimposed on the screen, and it was gazing directly at or away from the participant. The authors observed larger differences between congruent and incongruent trials (i.e., the Flanker effect) in the direct compared to the averted gaze condition, suggesting an increased interference caused by mutual gaze with the robot. Similar results have been also reported by Ciardo and Wykowska (2022). The authors showed that eye contact and avoiding the gaze of a humanoid robot can modulate cognitive conflict experience in a subsequent task. Specifically, participants were asked to perform an auditory Simon task while looking at a humanoid robot placed in front of the participants. At the end of each trial, following participants’ responses, the robot established eye contact or avoided gaze with the participants, irrespectively of their actual performance. Results showed that following eye contact, participants experienced more cognitive conflict, i.e., the SE was larger in magnitude. In line with Kompatsiari et al. (2022), these studies suggest that mutual gaze in HRI can make conflict monitoring less efficient. Eye contact with a robot was also suggested to make it more difficult to divert attention from a cued location (i.e., attentional disengagement; Ciardo & Wykowska, 2022; Kompatsiari et al., 2022), and to prolong the time needed to make decisions (Belkaid et al., 2021). Notably, the negative effect of eye contact on the inhibition of task-irrelevant information has been observed regardless of whether the social signal was used as a cue (Kompatsiari et al., 2022) or as feedback, affecting performance in subsequent trials (Ciardo & Wykowska, 2022).

### Robots as competitors

One possible explanation for why eye contact influences cognitive conflict in HRI is that it might make users feel monitored, eliciting negative feelings associated with a sense of threat (e.g., Stanton & Stevens, 2017). Indeed, the presence of robots may elicit negative emotions also if the robot is presented as a competitor, rather than as a team member. Spatola and colleagues (2018) explored the role of humanoid robots in a competitive, instead of a cooperative, scenario. The authors asked participants to complete a Stroop task alone, then again while a humanoid robot that they interacted with previously in a positive or negative way was standing next to them to monitor their performance. They found that the Stroop interference was reduced when participants were monitored by a robot with which they had a negative interaction compared to a robot that they positively interacted with, or when they were not monitored at all. This result seems to suggest that when a robot is physically present to monitor a human’s performance, without sending social signals and being perceived as an opponent instead of a team member, response selection improves. In the context of a go/no-go task (Donders, 1868; 1969, see Table 1), Currie and Wiese (2019) investigated motor inhibition when participants played against a preprogrammed robot or a robot that they thought was controlled by a human. In their setup, participants and the robot (Cozmo, Digital Dream Labs, Anki Robotics) sat facing each other with one LED block in front of the participant and another in front of the robot. Their task was to tap the block as fast as possible when the LEDs on the block lit up, but withhold their tap when it lit up with the color red. They found that participants had higher false alarms on no-go trials (i.e., they acted when they were not supposed to do it) and higher hit rates on go trials, indicating that when participants thought the robot was human-controlled, response inhibition was less efficient (Currie & Wiese, 2019). The inconsistencies observed in this literature suggest that further evidence is needed to understand how robots modulate cognitive conflict in scenarios in which robots are acting as supervisors or competitors (i.e., possibly eliciting negative emotions in the human partner).

## Guidelines for the use of humanoid robots for cognitive offloading

Across this review of selected literature, we explored how robots can offload humans on different cognitive conflict tasks, including performing joint tasks, having robots deliver social cues, or having robots act as supervisors or competitors. What emerges from previous research is that teaming up with robots is not always beneficial. Some factors related to the robot (e.g., Ciardo & Wykowska, 2022; see guidelines 1, 2, and 3) and to the task (e.g., Currie & Wiese, 2019; see guidelines 4 and 5) could affect the efficacy of the collaboration with robots in daily routines (see also Natarajan et al., 2023, for other important challenges). Based on these findings, we will provide guidelines for the optimal use of robots in collaborative cognitive tasks, with the goal of supporting the use of robots as cognitive offloading tools.

1. *Take advantage of a robot’s congruent social signals*. A large body of research in HRI showed that social signals delivered by robots are attended even if task-irrelevant (e.g., Ciardo & Wykowska, 2022; Kompatsiari et al., 2022; Perez-Osorio et al., 2021). As discussed, the consequences are not always beneficial in laboratory settings, with incongruent gaze cues distracting participants. However, the implications for real-world applications can be promising. First, robots used in real-world contexts, such as industry, education, or health care, can be programmed to use social signals, such as gaze, only in specific situations in which the congruency with relevant to-be-attended locations can be controlled. For example, in educational settings, the robot could use gaze cuing to redirect children’s attention to a task after distractions. In this case, the gaze could be directed only toward task-relevant locations or items (i.e., congruently). Not only would this have short-term effects, offloading cognitive processes, but long-term exposure to a robot companion that sends reliable cues would also increase humans’ beliefs about a robot’s expertise, eventually enhancing the likelihood of humans choosing the robot itself for cognitive offloading in the future (Weis & Wiese, 2022).

Second, the fact that robots can facilitate performance via appropriate social signals is also important for a cost-related reason. It is yet true that robots are becoming more and more used in many workplaces; however, they still come at a very high cost and thus, they are still currently prevalent in companies that have important capital to invest in. Since signals like gaze cuing do not necessarily need an embodied robot to be delivered (Spatola & Huguet, 2021), companies could use current knowledge to implement similar tailor-made virtual assistants, at a relatively low production cost (compared to embodied robots). This is intriguing because it would represent an affordable solution to include robots even in small work settings. We are already used to interacting with virtual assistants (many of us may remember "Clippy" the paperclip, one of the first and most popular Microsoft Office's virtual assistants). Clippy was created thanks to studies observing that humans had more positive experiences with technology if technology showed physical human-like features (Nass & Reeves, 1997). Many of us could also remember that attempt failed to persuade users and that Clippy was perceived as frustrating by most of the users (e.g., Iftikhar et al., 2023). A possible reason why this tool failed to be perceived as helpful might rely on its lack of social intelligence and dynamicity (Baym et al., 2019; Iftikhar et al., 2023; Nass & Yen, 2010). For example, it could not learn a user’s name or preferences (Kao et al., 2020) and its answers to the users were often defined as “rude” (e.g., Iftikhar et al., 2023). Importantly, since this first attempt, research has made great strides, and we can better understand the robot’s or setting’s features that can make the relationship successful.

2. *Build robots that can adapt to a dynamic environment*. One important factor for effective HRI is the robot’s ability to engage in social dynamics and to adapt its behavior to a social setting. For instance, being able to adapt to a dynamic environment is important to build trust in robotic agents (e.g., Chiou & Lee, 2023). Trust has been defined as the perception of the degree of aid that a robotic agent can provide in achieving a goal under conditions of uncertainty (Lee & Moray, 1992; Lee & See, 2004), and it does not only depend upon competency, but also on a robot’s interpersonal social skills (e.g., Stower et al., 2021). Previous research pointed out that trust in robotic systems is essential for efficient collaboration (Freedy et al., 2009; Billings et al., 2012; Hanckok et al., 2011; Lewis et al., 2018). The level of trust toward the robotic system seems to also have an important impact on the subjective perception of cognitive load (Ahmad et al., 2019; Baltrusch et al., 2022; Freedy et al., 2007), since it has been shown that high trust ratings decrease the perception of cognitive load in human users (Ahmad et al., 2019). In general, trust seems to foster cooperation and reliance, making individuals more receptive to the supportive role of humanoid robots across various tasks. According to the model proposed by Chiou and Lee (2023), trust is described as a dynamic construct that depends on the interdependent interaction between humans and automated systems, rather than being a static concept that needs to be trained. According to this framework, trust is not only a matter of reliance or reliability of robots, but other factors related to a robot’s social presence can affect trust, and consequently the success of human–robot collaboration. In line with what we said about Clippy, social robots need to be adaptive and “aware” to engage in successful social interactions, meaning not only that we need to perceive their intentionality, but also that we need to feel like they can adjust their behavior based on evolving mutual goals. Trust is more difficult to be promoted when robots can just perform predefined tasks applying a set of rules that are valid in a structured environment (Chiou & Lee, 2023), simply because people do not act in structured environments, as interruptions or unexpected events are normal in everyday environments. Based on these insights, a set of design features could be developed to maximize the likelihood of humans engaging in satisfying social interactions with robots (Fong et al., 2003; Krueger & Wiese, 2021). Many robots are static in their behavior, which means that when the operator moves away from the robot platform, it is not very autonomous in how it engages the environment around it (Inagaki et al., 2003; de Visser & Parasuraman, 2011; Sheridan, 2016). If we design a robot to perform task A in context B, it would not automatically know how to do task A in a different context, C. We propose using research to create more autonomous robots, as recent studies in adaptive automation show this could be a promising solution (Kaber, 2017; Kohn et al., 2021; Krueger & Wiese, 2021; Calhoun, 2022).
3. *Avoid prolonged eye contact with robots.* From the analysis of previous findings (Belkaid et al., 2021; Ciardo & Wykowska, 2021; Kompatsiari et al., 2022; Spatola et al., 2018), mutual gaze (i.e., eye contact) during HRI may have negative effects on cognitive load (i.e., increased cognitive conflict experienced in the eye contact condition; Spatola et al., 2018) as well as other processes such as decision-making and attention (Belkaid et al., 2021; Kompatsiari et al., 2022). The literature about affective reactions to mutual gaze during human–human interaction provided inconsistent findings, with studies using self-assessment tools showing aversive reactions to mutual gaze and studies using objective measurements (i.e., behavior or physiology rather than self-reports) showing more positive responses (Lawson, 2015; see Hietanen, 2018, for a review). One possible explanation could be that humans have an inherent need for social inclusion (e.g., Maslow, 1943) and that mutual gaze signals interest and inclusion (Wirth et al., 2010), thus triggering positive feelings, but that participants report aversion due to other biases (Hietanen, 2018). The situation, however, could be different in HRI. The need for social inclusion could be minimal and mutual gaze could be associated with a sense of being monitored, making participants feel threatened rather than included in a group by the robot (Stanton & Stevens, 2017). Teaming up with robots might raise feelings of both excitement and fear about these systems' autonomy, and the concerns about the potential replacement of humans with autonomous systems may be particularly intense if robots are included in non-joint tasks, in which humans could feel displaced and/or monitored by the robot. For example, the attitude toward social robots and other interactive autonomous systems received significant attention from the ethics communities, with important discussions on the societal impact of technological development (e.g., de Pagter, 2023). It should be noted that a recent study (Kiilavuori et al., 2021) found similar effects of eye contact with robots and other humans on emotional and attentional psychophysiological indexes (i.e., indexing positive reactions to eye contact with the robot), suggesting that participants were ascribing mental attributes to robots, in line with studies showing that humans can attribute mental states (i.e., intentionality) to robots (e.g., Gazzola et al., 2007; Hofree et al., 2015; Kiesler et al., 2008). Taken together, these findings suggest that humans' reactions to a robot's mutual gaze are complicated and probably multifaceted, thus requiring further investigations.
4. *Include robots if part of the main task can be entirely offloaded to them.* Whether or not collaborating with robots is going to be beneficial to task performance may depend on the type of task that needs to be completed. In some of the tasks we reviewed (Bunlon et al., 2018; Currie & Wiese, 2019), the cognitive load was not reduced because participants could not offload part of the main task to the robot, as the robot was not taking over part of task demands. Other scenarios might require a more efficient division of the work, in which humans can ignore information attended by the robot (Wahn et al., 2018). In visuospatial attention or perceptual decision-making tasks, the load can be divided between participants because these types of tasks usually allow for each participant to attend to part of the information and ignore the rest, since the other participant can attend to it (Wahn et al., 2023). In the context of a human-algorithm collaboration, Wahn and colleagues (2023) explored the extent to which humans offloaded parts of a high-demand attentional task to an algorithm. They asked participants to perform a multiple object tracking task (MOT; Pylyshyn & Storm, 1988), in which participants had to monitor a set of moving target items on a computer screen, ignoring surrounding distractors. Typically, humans can monitor a maximum of four objects (Intriligator & Cavanagh, 2001), but in the joint condition, participants had the opportunity to offload a subset of to-be-monitored items to the algorithm. The authors showed that participants decided to monitor a smaller number of targets in the joint condition, and this improved their overall performance (i.e., they committed fewer errors). Thus, collaboration in HRI may be most efficient when robots take over parts of a task, allowing their algorithms to improve accuracy by reducing the workload on humans.
5. *Beware of how prior interactions with a robot may impact task learning.* Many instances require humans to learn a novel task. While it may be easy to include an artificial interaction partner (i.e., a robot) in the task sequence, it may not always be beneficial. A study (Abubshait et al., 2021b) suggested that different degrees of familiarity with a robot (assigned via length of prior interactions) modulate the learning of reward information in a gambling task. In their experiment, participants started with a familiarization phase and were divided into three groups: one group was asked to play with a robot^1^ that they have not seen before during a single laboratory session of about 20 min (short interaction condition), a second group took the robot home and played with it for five consecutive days (long interaction condition), and a last group of participants did play with the robot at all (no familiarization/control condition). After this first familiarization phase, all participants performed the main task of interest, namely a learnable gambling task, where they learned the contingencies of “winning” and “losing” by choosing one of two colors (e.g., blue and green), with each color being associated with a different probability of winning. In this phase, the robot was present but not interacting in any way with the participant. After choosing one of the two colors, they received feedback on the screen on whether they won or lost the trial, then would have to choose again. Their results showed improved learning in the long interaction group compared to the short interaction group, but best learning rates for the control group (for a replication of the study using reward-elicited Event-Related Potentials see, Abubshait et al., 2021a). These results suggest that prior interaction with a robot can alter reward-based learning—a process that supports automatic attentional selection of important stimuli (e.g., Anderson, 2016; Gong & Li, 2014), alleviating the overall load imposed by the novelty of task—with more efficient learning in the control condition (i.e., no familiarization with the robot) compared to both interaction conditions, as participants were able to learn the contingencies of winning or losing in the gambling task using feedback fastest (see also Kalyuga, 2013; Kirschner et al., 2018, for similar discussions in instructional design). Whereas further studies are needed to understand how prior interactions with a robot shape learning processes, it could be speculated that the control group had better learning rates because they combined the new task rules and a new companion as a single to-be-learned contingency. In addition, it is possible that the control condition did not alleviate cognitive conflict, but that a "novelty effect" (see also “Recommendations for future research”) present in the short interaction group depleted more cognitive resources, which may have attributed to the slower learning rates. This is possible as slower learning rates were not shown in the group experiencing repeated interactions.

## Limitations of the current work

In the current work, we reviewed a selection of studies that we believe provide important insights about how to improve robots' efficacy in reducing cognitive load and people's acceptance of robots as potential allies in their daily routines. We understand that the selection of studies included is not comprehensive because of the great abundance of research that investigated the role of robots in cognitive tasks. To begin with, we decided to focus on studies exploring the effects of HRI on standardized cognitive conflict tasks. Many studies employed behavioral measures to evaluate cognitive load during HRI, targeting task completion time, error rates, and reaction times while performing single (Jou et al., 2009) and dual tasks (Chen & Joyner, 2009; Hopko et al., 2021), but did not use standardized paradigms. While we believe that other non-standard paradigms can be informative, standardized tasks allow for a more precise exploration of the cognitive processes that underlie specific tasks, and this could lead to an easier generalization of the findings. We also did not include evidence coming from studies using physiological signals such as eye movements, skin conductance, heart rate, and brain activity as indicators of the amount of cognitive load in HRI (Palinko & Sciutti, 2014; Kruger & Doherty, 2016; Memar & Esfahani, 2018; Novitzky et al., 2018). Analyzing responses from the body and the brain allows having a direct measure of load, but physiological measures are more expensive to collect, and their collection is rarely feasible in real-world contexts. For this reason, we focused on tools that are more suitable for real-world settings and that could provide more informative data (i.e., giving direct information about performance modulations).

Subjective tools, such as self-reports, are also extensively used to explore cognitive load during human–robot collaborations. We decided to focus on objective and standardized measures for a more robust analysis of cognitive dynamics subtending human–robot interactions. Moreover, subjective measures are susceptible to several limitations. Individuals may have difficulty articulating or being fully aware of their cognitive states, leading to potential biases or inaccuracies in their subjective reports (e.g., Hietanen, 2018), and individual and cultural influences can impact the reliability and generalizability of findings (e.g., Johnson & Widyanti, 2011; Westbrook et al., 2013). Finally, many scales used in HRI are not validated and are tested on inadequate sample sizes (e.g., Ötting et al., 2022; Stower et al., 2021; Yuan et al., 2021), making it even more complicated to navigate the myriads of tools currently available. Even though subjective tools have not been included in the present contribution, we believe that their integration with objective and standardized measures could allow researchers to obtain a comprehensive understanding of the cognitive phenomena involved during HRI.

On the other hand, standardized cognitive conflict tasks still face several limitations. First, they mimic but rarely represent what happens in real-world contexts. Participants could be asked to perform a typical task that is aimed at isolating a cognitive process (e.g., a stop-signal task to isolate response inhibition) in a short amount of time. They could be instructed to achieve the best performance while being in a controlled environment (e.g., silent, quiet, in optimal lighting conditions, etc.) where failures have almost no consequences. This is very far from what happens in real-world scenarios, in which a loss of control could cause dramatic outcomes and situations are not standardized and controlled (e.g., one should flexibly react to unexpected events, or to other people’s errors, etc.; Hoc & Amalberti, 2007). Second, most of the studies were characterized by a predominance of WEIRD (Western, Educated, Industrialized, Rich, Democratic) participants (Esterwood et al., 2021; Leichtmann et al., 2022; Stower et al., 2021). This could impact the reliability of results since cultural factors were observed to have a strong impact on HRI (Lim et al., 2021; Marchesi, Roselli, & Wykowska, 2021), and this lack of variety in samples might have a large impact on the observed results. Improving the quality of research methods would allow us to observe more reliable results and enhance our knowledge about the consequences of HRI on human cognition, eventually reducing the risk of wasting resources (e.g., using robots in inappropriate ways and/or contexts), increasing people’s mistrust of robots (e.g., people see that robots are not helpful, and become more skeptical about their use), and thus missing an opportunity to reduce the burdens of cognitive load.

Finally, other key factors have been suggested as potential reasons for the limited success of the collaboration with robots but were not mentioned here. Further studies are needed to systematically explore their effects and interactions in HRI contexts. These factors include: societal attitudes toward automation, with individualistic orientation and societies reacting with skepticism or fear toward robots limiting the positive outcomes of human–robot collaboration in general (e.g., Lim et al., 2021; Marchesi et al., 2021); the robot's appearance (i.e., anthropomorphism), with more socially engaging or relatable robots being more likely to elicit social facilitation effects (Duffy, 2003); variations in individual traits, such as social anxiety, familiarity with technology, and attitudes toward robots, with individuals responding positively to robots more probably experiencing social facilitation during HRI (e.g., Nomura et al., 2006, 2008); the quality of the feedback that the robot send back to the human: if the feedback provided by the robot is inaccurate, delayed, or ambiguous, it can lead to confusion rather than facilitation (e.g., Fischer et al., 2013); and task complexity, with increased social facilitation effects when the task is simple and well known (e.g., Riether et al., 2012). Critically, we do not know how much the effects are generalizable to different settings. Systematic investigations of all these moderating factors might allow us to gain valuable insights into the design and implementation of social humanoid robots that can truly alleviate cognitive overload and enhance human performance in diverse workplace settings. In the following section, we will introduce some general recommendations that we believe could help researchers to advance the theoretical and applied knowledge in HRI, by supporting the reliability and replicability of results.

## Recommendations for future research

1. *Use established benchmark experiments.* To systematically explore what modulates cognitive offloading in HRI results need to be replicable and scientifically and theoretically grounded. Something that could help could be using standardized paradigms like the ones described in Table 1 or developing new paradigms that could be used systematically and adapted in different HRI contexts. Following what we already introduced in the previous Section, however, we recommend researchers create experimental setups for their standardized laboratory tasks that are representative of real-world scenarios. For example, the environment could have realistic acoustic and lighting conditions or include unexpected events or outcomes (such as rewards and/or losses comparable to those experienced in real life). In addition, we would like to reiterate that researchers should include participants coming from more varied backgrounds, to provide results that are inclusive of different populations (i.e., not representing only WEIRD populations; Stower et al., 2021).
2. *Attenuate the novelty effect with longitudinal designs.* Whenever participants enter a social HRI laboratory, they have to share their space with a social partner that is novel to them. They are not acting in a well-established context, as they do not have conceptual knowledge about that situation. The incoming event (i.e., interacting with a robot) does not match the contents in their memory (Smedegaard, 2022; Tulving & Kroll, 1995). This mismatch elicits cognitive processes that are not activated during more familiar contexts, such as the disruption of habitual schemes, the processing of unexpected events, and the creation of a new alternative routine (Smedegaard, 2022). As we gain experience with a certain situation, we get to know how to best make sense of it. Psychological research suggests that, once we create a new stimulus–response association, we also activate cognitive processes aimed at justifying that association, and this could affect our attitude toward that stimulus (Harmon-Jones et al., 2015). Accordingly, if participants are interacting with a robot for the first time (and this plausibly was the case for many participants in HRI studies), their attitudes and feelings about the robot may be driven by a mere novelty effect. Of course, the state of uncertainty that results from interacting with these new agents is only temporary. Research should, on the one hand, explore how novelty affects the way participants engage with the robot, and eventually weigh current evidence based on that. On the other hand, more longitudinal studies should address how participants' interactions with a robot change as a function of familiarity with the robot. This would allow researchers to avoid confounding factors related to novelty (Smedegaard, 2022), and to systematically explore how familiarity with HRI scenarios modulates cognitive processes (Abubshait et al., 2021a, 2021b)
3. *Utilize multimodal metrics.* An approach that has gained relevance in recent years in HRI is multimodal HRI. It refers to how humans can communicate with robots using many tools that pertain to different senses, such as speech, sounds, body or eye movements, and physical contact (Su et al., 2023). Our review of selected studies focused mostly on how robots can offload the processing of visual information during perceptual cognitive tasks because most of the research traditionally focused on this modality. However, different modalities can contribute to improving the quality of HRI by making the robot more socially competent and the interaction more intuitive for humans (Su et al., 2023), and this potentially facilitates cognitive offloading by humanoid robots. For example, feedback delivered by a robot can be more salient if it combines multiple modalities (Andronas et al., 2021). As Su and colleagues (2023) mentioned, a robot can use a combination of modalities for several purposes, such as signaling the status of the robot, pointing out a mistake in a given task (e.g., via a warning sound combined with a flashing light), or just having a natural communication with the human companion. Further research is needed to understand how to combine different modalities to facilitate the use of humanoid robots as cognitive offloading tools.
4. *Lean toward open science and studies comparisons.* Related to what we mentioned about the need for more benchmark experiments, we cannot think about reproducing results in HRI without talking about transparency and open science (Romero, 2019). Researchers should provide more information on the design, testing procedures, and statistical analyses, but also make original materials, like codes and results publicly available (Maxwell, 2004). This would improve the replicability of studies and help researchers to better contextualize results (Leichtmann et al., 2022). For example, robots vary across studies, and having the source codes and data available may provide important information about the role that a specific robot's features play in observing certain effects. Transparency would also facilitate future researchers to compare the effects of a given variable on different measurements and experimental setups (e.g., does receiving ambiguous feedback from a robot similarly affect RTs and subjective measures of cognitive load?; Fischer et al., 2013).

## Conclusions

Relying on technology to complete cognitive tasks is becoming increasingly common due to the availability of tools for cognitive support and is crucial for freeing up cognitive resources and reducing cognitive load (e.g., Clark, 2011). Throughout this contribution, we explored the potential use of humanoid robots for cognitive offloading. Addressing how humanoid robots can impact cognitive load during HRI is important if we want to create workplaces where people can work efficiently side by side with robots. Based on the reviewed studies, we suggest that humanoid robots do not always contribute positively to offloading cognitive control in humans. When the human and robot share tasks to reach a common goal, the collaboration comes at a cost in terms of load and performance. However, humanoid robots can still be helpful by delivering appropriate social signals, for example, cuing task-relevant locations, making the inclusion of humanoid robots in real-world scenarios promising. An important goal for future research would be to integrate the current knowledge into new protocols aimed at including robots in the most functional way in real-world settings.

## Data Availability

Not applicable.

## Abbreviations

CE: Commission errors
CRT: Correct detection reaction times
HRI: Human-robot interaction
JA: Joint action
‍MOT: Multiple object tracking task
OE: Omission errors
RTs: Reaction times
‍SE: Simon effect
SSD: Stop-signal delay
SSRT: Stop-signal reaction time
WEIRD: Western, Educated, Industrialized, Rich, Democratic

## Footnotes

1 The robot that was used in this experiment was Cozmo (Digital Dream Labs, Anki Robotics), an educational toy robot. The games that people can play with were all preprogrammed by the developers of the robot. The games included a variation of a color-based go-no-go task where the robot and the participants played against one another. This robot has been used in the past in many human–robot interaction studies. For a description of the metrics from the games, please see Hortensius et al. (2022).
